# Sanitary Legislation

**Published:** 1857-10

**Authors:** 


### SANITARY LEGISLATION.

The Board of Health continues; and the Right Hon. W. F. Cowper has again been appointed its President. Medical Reform is settled negatively for awhile. Mr. Gamgee, not satisfied with the conduct of the Home Secretary, has had a fresh brush with Lord Palmerston, and has improved the Premier considerably. In two addresses to his lordship on "Medical Reform a Social Question", Mr. Gamgee, breathing out burning vapour of alcohol, and flourishing the trident of liberty, equality, and fraternity in physics, proclaims a great many solemn truths, which would have come out more forcibly if they had been given with less zeal. His great argument (and who shall deny it?) is, that medical reform lies with medicine as a profession, not with parliament; that it must be founded on science, not on politics.

Lord Elcho has also published a speech on Medical Reform.

An important movement is in progress, viz., the formation of a society called the "National Association for the Promotion of Social Science." The objects are excellent, but the title unfortunate, we fear ruinous. Lord Brougham is the president, and the first meeting will be held at Birmingham on October 12th, and the four following days. The subjects for consideration have been divided into five departments, viz., Jurisprudence and Amendment of the Law, Education, Punishment and Reformation, Public Health, Social Economy. We wish the society every possible success; its designs are great, and its results, whether in the way of success or of failure, will be extreme.

### REPORTS AND RETURNS.

Crimean Sanitary Commissioners' Report.—The Report of the Sanitary Commissioners sent out to the Crimea forms a handsome work, illustrated with beautiful maps and drawings.

Sir John Hall has replied to this report, and defended himself and his colleagues from the slur which, as he thinks, has been thrown over him and them by the commissioners.

Report on Sheep and Contagious Diseases Prevention Bill.—This report is valuable for the able evidence by Professor Simonds on the subject of contagion. The committee draw no conclusions of any length or importance; for which they are commendable, as the questions before them were far too complicated for the decision of unprofessional men. This with all respect. The evidence of Mr. Miles as to the indifferent inspection of Newgate Market is confirmatory of the worst statements of Mr. Gamgee.

Netley Hospital.—A return relating to the construction of the Netley Hospital, exhibits a ridiculous specimen of the contradictions of authorities in reference to common sense arrangements. A great hospital for sick soldiers, to be called the Royal Victoria Hospital, being in course of construction at Netley, the medical officers of the Middlesex Hospital, struck with the deficient contrivances for supplying sick soldiers with their daily air-food, sent in a protest to Lord Panmure, and a recommendation in support of the plan on which the Bordeaux Hospital is built. That is to say, they suggested for the Netley Hospital separate wards, open to the air and light on each side, in preference to wards intersected within by a vast corridor, or receptacle for every impure emanation. Lord Panmure upon this remonstrance made further inquiries, and a modification of the original plan of the hospital was drawn up, but not to the satisfaction of the Middlesex authorities, who still stick to their point manfully, and deserve the best thanks of all sensible sanitarians. It is quite hopeless, however, to attempt any defence of the position of these gentlemen; to the unbiassed their arguments are a demonstration, to the biassed even a demonstration is a waste of brain-matter. Time, and the mortality of the Netley Hospital, must be left as arbitrators.

"The life and death test
Is the best, is the best."

